# The Human Genome Project

**Published:** 1995

**Authors:** Francis S. Collins, Leslie Fink

**Affiliations:** Francis S. Collins, M.D., Ph.D., is director and Leslie Fink is communications officer at the National Center for Human Genome Research at the National Institutes of Health, Bethesda, Maryland

**Keywords:** genome, genetic mapping, DNA, applied research, molecular genetics

## Abstract

The Human Genome Project is an ambitious research effort aimed at deciphering the chemical makeup of the entire human genetic code (i.e., the genome). The primary work of the project is to develop three research tools that will allow scientists to identify genes involved in both rare and common diseases. Another project priority is to examine the ethical, legal, and social implications of new genetic technologies and to educate the public about these issues. Although it has been in existence for less than 6 years, the Human Genome Project already has produced results that are permeating basic biological research and clinical medicine. For example, researchers have successfully mapped the mouse genome, and work is well under way to develop a genetic map of the rat, a useful model for studying complex disorders such as hypertension, diabetes, and alcoholism.

The Human Genome Project is an international research project whose primary mission is to decipher the chemical sequence of the complete human genetic material (i.e., the entire genome), identify all 50,000 to 100,000 genes contained within the genome, and provide research tools to analyze all this genetic information. This ambitious project is based on the fact that the isolation and analysis of the genetic material contained in the DNA^1^ (figure 1) can provide scientists with powerful new approaches to understanding the development of diseases and to creating new strategies for their prevention and treatment. Nearly all human medical conditions, except physical injuries, are related to changes (i.e., mutations) in the structure and function of DNA. These disorders include the 4,000 or so heritable “Mendelian” diseases that result from mutations in a single gene; complex and common disorders that arise from heritable alterations in multiple genes; and disorders, such as many cancers, that result from DNA mutations acquired during a person’s lifetime. (For more information on the genetics of alcoholism, see the articles by Goate, pp. 217–220, and Grisel and Crabbe, pp. 220–227.)

Although scientists have performed many of these tasks and experiments for decades, the Human Genome Project is unique and remarkable for the enormity of its effort. The human genome contains 3 billion DNA building blocks (i.e., nucleotides), enough to fill approximately one thousand 1,000-page telephone books if each nucleotide is represented by one letter. Given the size of the human genome, researchers must develop new methods for DNA analysis that can process large amounts of information quickly, cost-effectively, and accurately. These techniques will characterize DNA for family studies of disease, create genomic maps, determine the nucleotide sequence of genes and other large DNA fragments, identify genes, and enable extensive computer manipulations of genetic data.

## Focus of the Human Genome Project

The primary work of the Human Genome Project has been to produce three main research tools that will allow investigators to identify genes involved in normal biology as well as in both rare and common diseases. These tools are known as positional cloning (Collins 1992). These advanced techniques enable researchers to search for disease-linked genes directly in the genome without first having to identify the gene’s protein product or function. (See the article by Goate, pp. 217–220.) Since 1986, when researchers first found the gene for chronic granulomatous disease^2^ through positional cloning, this technique has led to the isolation of considerably more than 40 disease-linked genes and will allow the identification of many more genes in the future (table 1).

Each of the three tools being developed by the Human Genome Project helps bring the specific gene being sought into better focus (see sidebar, pp. 192–193). The first of these tools, the *genetic* map, consists of thousands of landmarks—short, distinctive pieces of DNA—more or less evenly spaced along the chromosomes. With this tool, researchers can narrow the location of a gene to a region of the chromosome. Once this region has been identified, investigators turn to a second tool, the *physical* map, to further pinpoint the specific gene. Physical maps are sets of overlapping DNA that may span an entire chromosome. These sets are cloned and frozen for future research. Once the physical map is complete, investigators will simply be able to go to the freezer and pick out the actual piece of DNA needed, rather than search through the chromosomes all over again. The final tool will be the creation of a *complete sequence* map of the DNA nucleotides, which will contain the exact sequence of all the DNA that makes up the human genome.

**Genetic Maps Provide Blueprint for Human Genome**

A primary focus of the Human Genome Project is to develop tools that will enable investigators to analyze large amounts of hereditary material quickly and efficiently. The success of this project hinges on the accurate mapping of each chromosome. The Human Genome Project is using primarily three levels of maps, each of which helps to increase understanding not only of the construction of individual genes but also of their relation to each other and to the entire chromosomal structure.

***Genetic Mapping***

Genetic mapping, also called linkage mapping, provides the first evidence that a disease or trait (i.e., a characteristic) is linked to the gene(s) inherited from one’s parents. Through genetic mapping, researchers can approximate the location of a gene to a specific region on a specific chromosome; the process is like establishing towns on a road map (figure 1). For example, Interstate 10 runs from Florida to California. It would be difficult to find a landmark along that highway if the only cities mapped were Jacksonville and Los Angeles. It would be much easier, however, to pinpoint the landmark if one knew that it was located between markers that are closer together (e.g., El Paso and San Antonio).

Genetic mapping begins with the collection of blood or tissue samples from families in which a disease or trait is prevalent. After extracting the DNA from the samples, researchers track linearly the frequency of a recurring set of nucleotides (represented, for example, by the letters “CACACA”) along a region of a chromosome. If this sequence is shared among family members who have the disease, the scientists may have identified a marker for the disease-linked gene. Mapping additional DNA samples from other people with and without the disease allows researchers to determine the statistical probability that the marker is linked to the development of the disease.

**Figure 1:**
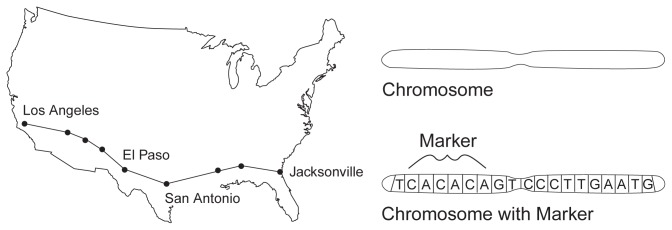
Genetic Map. Just as locating a landmark on a particular highway is easier if one can narrow the area of the search to between two nearby points, or markers (e.g., El Paso and San Antonio on Interstate 10), researchers first try to narrow their search for particular genes to a segment of chromosome denoted by a specific sequence of nucleotides (e.g., CACACA).

***Physical Mapping***

**Figure 2:**
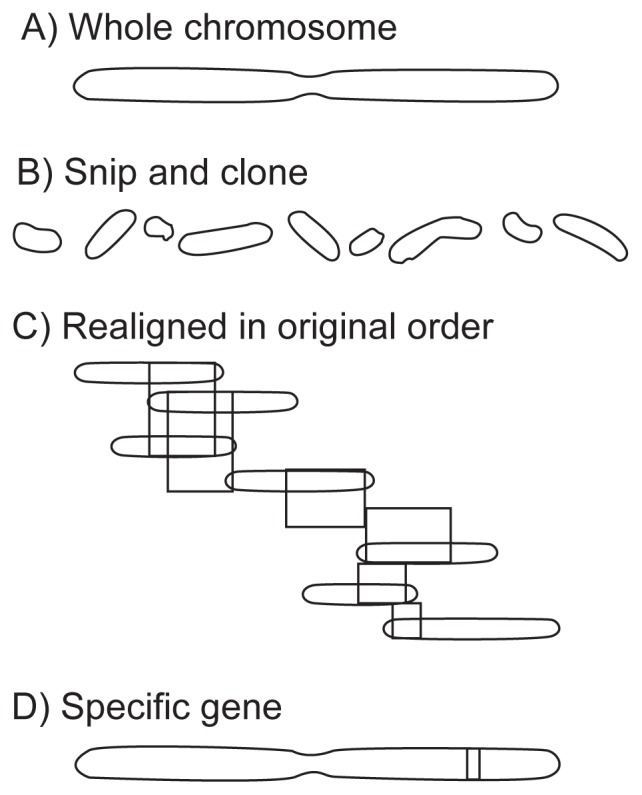
Physical Map. Using various methods, A) whole chromosomes are B) snipped into large fragments of DNA (i.e., sequences of nucleotides) and then cloned. C) These cloned DNA pieces then are realigned in the order in which they originally occurred in the chromosomes and stored. The stored pieces can be used for further studies such as D) finding specific genes.

Physical mapping generates sets of overlapping DNA fragments that span regions of—or even whole—chromosomes. These DNA fragments, which can be isolated and stored for future analysis (figure 2), serve as a resource for investigators who want to isolate a gene after they have mapped it to a particular chromosome or chromosomal region. The physical map allows scientists to limit the gene search to a particular subregion of a chromosome and thus zero in on their target more rapidly.

One early goal of the physical mapping component of the Human Genome Project was to isolate contiguous DNA fragments that spanned at least 2 million nucleotides. Considerable progress has been made in this area, with sets of contiguous DNA fragments (“contigs”) now frequently ranging from 20 to 50 million nucleotides in length. Because the order of DNA fragments in a physical map should reflect their actual order on a chromosome, correct alignment of contigs also requires a set of markers to serve as mileposts, similar to those of an interstate highway. Genome scientists have developed a physical map that currently contains about 23,000 markers, called sequence tagged sites (STS’s). Scientists likely will meet their ultimate goal of establishing 30,000 STS markers on the physical map—one every 100,000 nucleotides—within the next year or two. This detailed STS map will allow researchers to pinpoint the exact location of any gene within 50,000 nucleotides of an STS marker.

**Figure 3:**
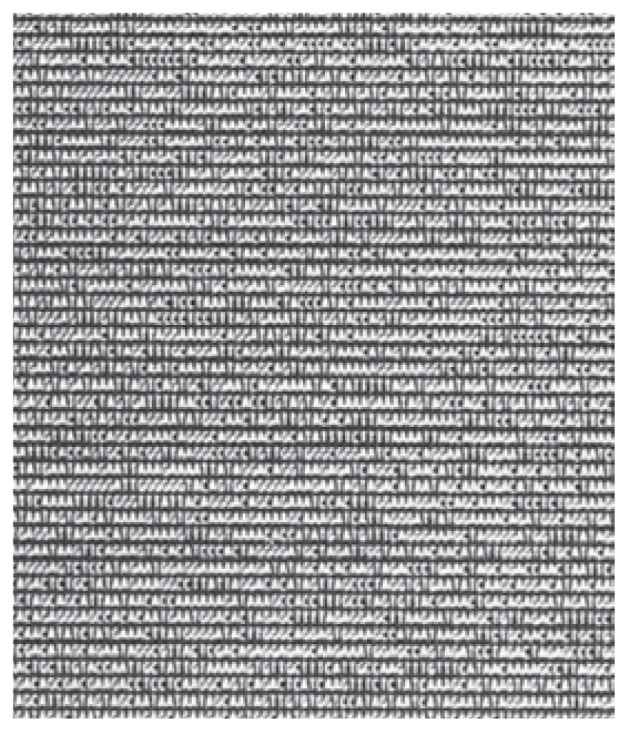
Part of the DNA sequence map of a virus containing 10,000 nucleotide bases. For comparison, the human genome contains approximately 3 billion nucleotide bases.

Researchers also are attempting to use fragments of expressed genes known as expressed sequence tags (EST’s), which are made from complimentary DNA, as markers on the physical genome map. By using EST’s, they hope to increase the power of maps for finding specific genes. A recent collaboration between Merck and Co. (a major pharmaceutical corporation) and researchers at Washington University in St. Louis, Missouri, will provide a resource for placing tens of thousands of such markers derived from actual genes on the physical map.

Marker development to be used in creating both the linkage and the physical maps also takes into account the need for connectivity between these two types of maps. Information learned from one stage of the gene-finding process must be easily translatable to the next.

***The DNA Sequence Map***

The Human Genome Project’s most challenging goal is to determine the order (i.e., sequence), unit by unit, of all 3 billion nucleotides that make up the human genome. Once the genetic and physical maps are completed, a sequence map can be constructed, which will allow scientists to find genes, characterize DNA regions that control gene activity, and link DNA structure to its function.

To date, the technology for this work has been developed and implemented primarily in model organisms. For example, researchers now have sequenced 25 million DNA nucleotides from the roundworm—about 25 percent of the animal’s genome—and, in the process, have increased their annual sequencing rate to 11 million nucleotide bases (figure 3). The investigators expect to finish sequencing the roundworm genome by the end of 1998. The complete DNA sequence of yeast and *E. coli* genomes will be determined even sooner.

—Francis S. Collins and Leslie Fink

To make all this information available to researchers worldwide, the project has the additional goal of developing computer methods for easy storage, retrieval, and manipulation of data. Moreover, because researchers often can obtain valuable information about human genes and their functions by comparing them with the corresponding genes of other species, the project has set goals for mapping and sequencing the genomes of several important model organisms, such as the mouse, rat, fruit fly, roundworm, yeast, and the common intestinal bacterium *E. coli*.

## Technological Advances in Genomic Research

The need for large-scale approaches to DNA sequencing has pushed technology toward both increasing capacity and decreasing instrument size. This demand has led, for example, to the development of automated machines that reduce the time and cost of the biochemical processes involved in sequencing, improve the analysis of these reactions, and facilitate entering the information obtained into databases. Robotic instruments also have been developed that expedite repetitive tasks inherent in large-scale research and reduce the chance for error in several sequencing and mapping steps.

Miniaturization technology is facilitating the sequencing of more—and longer—DNA fragments in less time and increasing the portability of sequencing processes, a capability that is particularly important in clinical or field work. In 1994, for example, the National Institutes of Health (NIH), through its National Center for Human Genome Research (NCHGR), began a new initiative for the development of microtechnologies to reduce the size of sequencing instrumentation and thereby increase the speed of the sequencing process. NCHGR also is exploring new strategies for minimizing time-consuming sequencing bottlenecks by developing integrated, matched components that will help ensure that each step in the sequencing process proceeds as efficiently as possible. The overall sequencing rate is only as fast as its slowest step.

Other developments in DNA sequencing have aimed to reduce the costs associated with the technology. Through refinements in current sequencing methods, the cost has been lowered to about $0.50 per nucleotide. Research on new DNA sequencing techniques is addressing the need for rapid, inexpensive, large-scale sequencing processes for comparison of complex genomes and clinical applications. Further improvements in the efficiency of current processes, along with the development of entirely new approaches, will enable researchers to determine the complete sequence of the human genome perhaps before the year 2005.

## Applications of the Human Genome Project

The detailed genetic, physical, and sequence maps developed by the Human Genome Project also will be critical to understanding the biological basis of complex disorders resulting from the interplay of multiple genetic and environmental influences, such as diabetes; heart disease; cancer; and psychiatric illnesses, including alcoholism. In 1994, for example, researchers used genetic maps to discover at least five different chromosome regions that appear to play a role in insulin-dependent (i.e., type 1) diabetes (Davies et al. 1994). Analyses to identify the genetic components of these complex diseases require high-resolution genetic maps and must be conducted on a scale much larger than was previously possible. Automated microsatellite marker technology^3^ now makes it possible to determine the genetic makeup (i.e., the genotype) of enough subjects so that genes for common diseases can be mapped reliably in a reasonable amount of time. NCHGR is planning a technologically advanced genotyping facility to assist investigators in designing research studies; performing genetic analyses; and developing new techniques for analyzing common, multigene diseases.

### Molecular Medicine

Efforts to understand and treat disease processes at the DNA level are becoming the basis for a new molecular medicine. The discovery of disease-associated genes provides scientists with the foundation for understanding the course of disease, treating disorders with synthetic DNA or gene products, and assessing the risk for future disease. Thus, by going directly to the genetic source of human illness, molecular medicine strategies will offer a more customized health management based on the unique genetic constitution of each person. Molecular medicine also will increase clinicians’ focus on prevention by enabling them to predict a person’s risk for future disease and offer prevention or early treatment strategies. This approach will apply not only to classical, single-gene hereditary disorders but also to more common, multi-gene disorders, such as alcoholism.

During the past 3 years, positional cloning has led to the isolation of more than 30 disease-associated genes. Although this number has increased dramatically, compared with the years predating the Human Genome Project, it is still a small fraction of the 50,000 to 100,000 genes that await discovery in the entire genome. NCHGR has helped develop efficient biological and computer techniques to identify all the genes in large regions of the genome. One technique was used successfully last year to isolate *BRCA1*, the first major gene linked to inherited breast cancer. The location of *BRCA1* first was narrowed to a DNA fragment of several hundred thousand nucleotides containing many genes. A process that isolates the protein-coding sequences of a gene (i.e., exon trapping) allowed researchers to identify and examine not only the correct *BRCA1* gene in that region but also several new genes that now serve as disease-gene candidates for future investigations.

### Diagnostics

Clinical tests that detect disease-causing mutations in DNA are the most immediate commercial application of gene discovery. These tests may positively identify the genetic origin of an active disease, foreshadow the development of a disease later in life, or identify healthy carriers of recessive diseases such as cystic fibrosis.^4^ Genetic tests can be performed at any stage of the human life cycle with increasingly less invasive sampling procedures. Although DNA testing offers a powerful new tool for identifying and managing disease, it also poses several medical and technical challenges. The number and type of mutations for a particular disease may be few, as in the case of achondroplasia,^5^ or many, as in the case of cystic fibrosis and hereditary breast cancer. Thus, it is essential to establish for each potential DNA test how often it detects disease-linked mutations and how often and to what degree detection of mutations correlates with the development of disease.

### Therapeutics

Gene discovery also provides opportunities for developing gene-based treatment for hereditary and acquired diseases. These treatment approaches range from the mass production of natural substances (e.g., blood-clotting factors, growth factors and hormones, and interleukins and interferons^6^) that are effective in treating certain diseases to gene-therapy strategies. Gene therapy is designed to deliver DNA carrying a functional gene to a patient’s cells or tissues and thereby correct a genetic alteration.

Currently, more than 100 companies conduct human clinical trials on DNA-based therapies (Pharmaceutical Research and Manufacturers of America [PRMA] 1995). The top U.S. public biotechnology companies have an estimated 2,000 drugs in early development stages (Ernst and Young 1993). Since 1988, NIH’s Recombinant DNA Advisory Committee has approved more than 100 human gene-therapy or gene-transfer protocols (Office of Recombinant DNA Activities, NIH, personal communication, April 1995). Seventeen gene-therapy products are now in commercial development for hereditary disorders, cancer, and AIDS (PRMA 1995).

## Ethical, Legal, and Social Concerns of the Human Genome Project

### Implications for Disease Detection

The translation of human genome technologies into patient care brings with it special concerns about how these tools will be applied. A principal arena in which psychosocial issues related to these technologies are being raised is the testing of people who may be at risk for a genetically transmitted disease but who do not yet show the disease’s symptoms (i.e., are asymptomatic). These concerns stem largely from the delay between scientists’ technical ability to develop DNA-based diagnostic tests that can identify a person’s risk for future disease and their ability to develop effective prevention or treatment strategies for the disorders those tests portend. In the meantime, people who undergo genetic tests run the risk of discrimination in health insurance and may have difficulty adapting to test results—particularly in families in which hereditary disease is common—regardless of whether a test indicates future disease. When no treatment is available and when no other medical course of action can be taken on the basis of such tests, the negative social, economic, and psychological consequences of knowing one’s medical fate must be carefully evaluated in light of the meager medical benefits of such knowledge.

To help ensure that medical benefits are maximized without jeopardizing psychosocial and economic well-being, the Human Genome Project, from its beginning, has allocated a portion of its research dollars to study the ethical, legal, and social implications (ELSI) of the new genetic technologies. A diverse funding program supports research in four priority areas: the ethical issues surrounding the conduct of genetic research, the responsible integration of new genetic technologies into the clinic, the privacy and fair use of genetic information, and the professional and public education about these issues.

Because of the many unresolved questions surrounding DNA testing in asymptomatic patients, in 1994 NCHGR’s advisory body released a statement urging health care professionals to offer DNA testing for the predisposition to breast, ovarian, and colon cancers only within approved pilot research programs until more is known about the science, psychology, and sociology of genetic testing for some diseases (National Advisory Council for Human Genome Research 1994). The American Society of Human Genetics and the National Breast Cancer Coalition have issued similar statements. More recently, the NIH–DOE [Department of Energy] Working Group on ELSI launched a task force to perform a comprehensive, 2-year evaluation of the current state of genetic testing technologies in the United States. The task force will examine safety, accuracy, predictability, quality assurance, and counseling strategies for the responsible use of genetic tests.

In a related project, NCHGR’s ELSI branch spearheaded a new group of pilot studies shortly after researchers isolated *BRCA1* and several genes for colon cancer predisposition. These 3-year studies are examining the psychosocial and patient-education issues related to testing healthy members of families with high incidences of cancer for the presence of mutations that greatly increase the risk of developing cancer. The results will provide a thorough base of knowledge on which to build plans for introducing genetic tests for cancer predisposition into medical practice.

### Implications for Complex Traits

Research in human genetics focuses not only on the causes of disease and disability but also on genes and genetic markers that appear to be associated with other human characteristics, such as height, weight, metabolism, learning ability, sexual orientation, and various behaviors (Hamer et al. 1993; Brunner et al. 1993). Associating genes with human traits that vary widely in the population raises unique and potentially controversial social issues. Genetic studies elucidate only one component of these complex traits. The findings of these studies, however, may be interpreted to mean that such characteristics can be reduced to the expression of particular genes, thus excluding the contributions of psychosocial or environmental factors. Genetic studies can also be interpreted in a way that narrows the range of variation considered “normal” or “healthy.”

Both reducing complex human characteristics to the role of genes and restricting the definition of what is normal can have harmful—even devastating—consequences, such as the devaluation of human diversity and social discrimination based on a person’s genetic makeup. The Human Genome Project must therefore foster a better understanding of human genetic variation among the general public and health care professionals as well as offer research policy options to prevent genetic stigmatization, discrimination, and other misuses and misinterpretations of genetic information.

## Progress on Genetic and Physical Maps

In the United States, NCHGR and DOE, through its Office of Environmental Health Research, are the primary public supporters of major genome research programs. In 1990, when the 15-year Human Genome Project began, NCHGR and DOE established ambitious goals to guide the research through its first years (U.S. Department of Health and Human Services and U.S. Department of Energy 1990). After nearly 6 years, scientists involved in the Human Genome Project have met or exceeded most of those goals—some ahead of time and all under budget. Because scientific advances may rapidly make the latest technologies obsolete, a second 5-year plan was published in 1993 (Collins and Galas 1993) to keep ahead of the project’s progress. Already, further technological advances make it likely that a new plan will be needed, perhaps as early as this year.

In 1994, an international consortium headed by the Genome Science and Technology Center in Iowa published a genetic map of the human genome containing almost 6,000 markers spaced less than 1 million nucleotides apart (Cooperative Human Linkage Center et al. 1994). This map was completed more than 1 year ahead of schedule, and its density of markers is four to six times greater than that called for by the 1990 goals. This early achievement is largely a result of the discovery and development of micro-satellite DNA markers and of large-scale methods for marker isolation and analysis.

In a related project, technology developed so quickly that a high resolution genetic map of the mouse genome was completed in just 2 years. NCHGR is now helping to coordinate an initiative with other NIH institutes, particularly the National Heart, Lung, and Blood Institute and the National Institute on Alcohol Abuse and Alcoholism, to develop a high-resolution genetic map of the rat, a useful model for studying complex disorders such as hypertension, diabetes, and alcoholism.

The original 5-year goal to isolate contiguous DNA fragments that span at least 2 million nucleotides was met early on; soon, more than 90 percent of the human genome will be accounted for using sets of overlapping DNA fragments, each of which is at least 10 million nucleotides long. Complete physical maps now exist for human chromosomes 21, 22, and Y. Nearly complete maps have been developed for chromosomes 3, 4, 7, 11, 12, 16, 19, and X.^7^

As the end of the first phase of the Human Genome Project draws near, its impact already is rippling through basic biological research and clinical medicine. From deciphering information in genes, researchers have gained new knowledge about the nature of mutations and how they cause disease. Even after someday identifying all human genes, scientists will face the daunting task of elucidating the genes’ functions. Furthermore, new paradigms will emerge as researchers and clinicians understand interactions between genes, the molecular basis of multigene disorders, and even tissue and organ function.

The translation of this increasing knowledge into improved health care already is under way; however, the value of gene discovery to the promising new field of molecular medicine will be fully realized only when the public is secure in the use of genetic technologies.

## Figures and Tables

**Figure 1:**
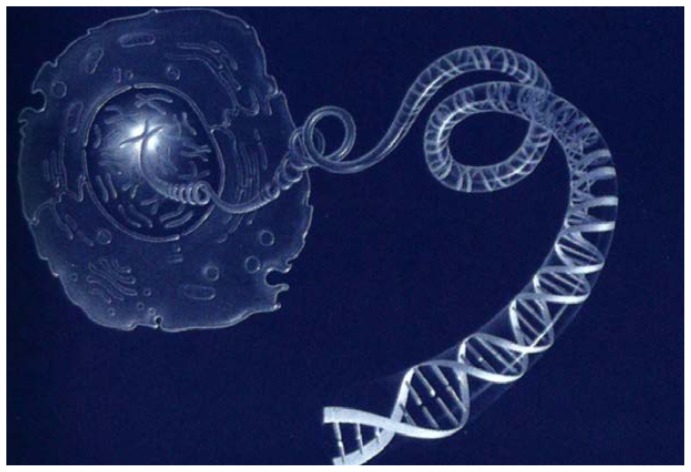
Artist’s rendering of the DNA molecule from a single cell.

**Table 1:** Disease Genes Identifed Using Positional Cloning

Year	Disease
1986	Chronic Granulomatous Disease Duchenne’s Muscular Dystrophy Retinoblastoma
1989	Cystic Fibrosis
1990	Wilms’ Tumor Neurofibromatosis Type 1 Testis Determining Factor Choroideremia
1991	Fragile X Syndrome Familial Polyposis Coli Kallmann’s Syndrome Aniridia
1992	Myotonic Dystrophy Lowe’s Syndrome Norris’s Syndrome
1993	Menkes’ Disease X-Linked A gammaglobulinemia Glycerol Kinase Deficiency Adrenoleukodystrophy Neurofibromatosis Type 2 Huntington’s Disease von Hippel-Lindau Disease Spinocerebellar Ataxia I Lissencephaly Wilson’s Disease Tuberous Sclerosis
1994	MacLeod’s Syndrome Polycystic Kidney Disease Dentatorubral Pallidoluysian Atrophy Fragile X “E” Achondroplasia Wiskott Aldrich Syndrome Early Onset Breast/Ovarian Cancer (*BRCA* 1) Diastrophic Dysplasia Aarskog-Scott Syndrome Congenital Adrenal Hypoplasia Emery-Dreifuss Muscular Dystrophy Machado-Joseph Disease
1995	Spinal Muscular Atrophy Chondrodysplasia Punctate Limb-Girdle Muscular Dystrophy Ocular Albinism Ataxia Telangiectasia Alzheimer’s Disease Hypophosphatemic Rickets Hereditary Multiple Exostoses Bloom Syndrome Early Onset Breast Cancer (*BRCA* 2)

## Footnotes

1 For a definition of this and other technical terms used in this article, see central glossary, pp. 182–183.

2 Chronic granulomatous disease is an inherited disease of the immune system.

3 Microsatellite markers are short DNA sequences that vary in length from person to person. The length of a particular marker is inherited from one’s parents, allowing researchers to track the markers through several generations of the same family.

4 For a recessive disease to develop, a person must inherit two altered gene copies, one from each parent. People who inherit only one altered gene copy usually are healthy (i.e., they do not show symptoms of the disease); these people are called asymptomatic carriers.

5 Achondroplasia is a disorder that results in defective skeletal development in the fetus and dwarfism. Affected children often die before or within their first year of life.

6 Interleukins and interferons are substances that stimulate and regulate the immune system.

7 Of the 23 chromosome pairs in human cells, 22 pairs are numbered according to their size, with chromosome 1 being the largest and chromosome 22 being the smallest chromosome. The gender-determining chromosomes are referred to as X and Y.
